# HfO_2_-Based Reconfigurable Radio Frequency Switches for All-Memristor Multistate Attenuator

**DOI:** 10.3390/nano16100605

**Published:** 2026-05-15

**Authors:** Yuanyuan Zhou, Yan Wu, Quan Yang, Weiran Cai, Xiaowei Zhang, Xiaolong Cai, Chenglin Du, Yuda Zhao

**Affiliations:** 1 College of Integrated Circuits, ZJU-Hangzhou Global Scientific and Technological Innovation Center, Zhejiang University, 38 Zheda Road, Hangzhou 310027, China; 2411100041@nbu.edu.cn (Y.Z.); 12560012@zju.edu.cn (Y.W.); 3210102046@zju.edu.cn (W.C.); 2 Faculty of Electrical Engineering and Computer Science, Ningbo University, Ningbo 315211, China; zhangxiaowei@nbu.edu.cn; 3 State Key Laboratory of Mobile Network and Mobile Multimedia Technology, Shenzhen 518055, China; du.chenglin@zte.com.cn; 4 ZTE Corporation, Shenzhen 518057, China

**Keywords:** non-volatile memristor, radio frequency switch, reconfigurable attenuator, hafnium oxide

## Abstract

Reconfigurable radio frequency (RF) attenuators are critical passive components for 5G-Advanced and emerging 6G wireless systems. Conventional tunable attenuators rely on solid-state switches combined with fixed resistor networks, which suffer from unavoidable static power consumption and severe parasitic degradation at high frequencies. Here, we systematically demonstrate HfO_2_-based non-volatile memristors as RF switches with tunable ON-state resistance (*R*_ON_), enabling a switching-attenuation-integrated multistate attenuator. The fabricated Au/HfO_2_/Ag devices exhibit stable bipolar resistive switching with an ON/OFF ratio exceeding 10^9^, reliable retention of 10^5^ s, and programmable *R*_ON_ continuously tuned from 5.8 Ω to 197.5 Ω. On-wafer RF characterizations from 10 MHz to 43.5 GHz reveal low insertion loss (−0.53 dB), high isolation (−26.8 dB), and clear scaling laws governing the effects of device geometry and *R*_ON_ on RF performance. Leveraging these unique characteristics, we propose a symmetric π-type programmable all-memristor attenuator architecture with a cascaded 2-unit configuration. The design achieves 12 discrete attenuation levels from 2 dB to 24 dB, a return loss better than 10 dB across the full band, and zero static power consumption without additional passive components or bias networks. This work establishes the fundamental material-device-RF performance relationship in HfO_2_-based RF switches and provides a compact, low-power, and highly integrable solution for next-generation reconfigurable RF front-ends.

## 1. Introduction

The rapid advancement of 5G-Advanced and upcoming 6G wireless communication systems has imposed urgent demands for high-integration, low-power, wideband, and reconfigurable radio frequency (RF) front-ends [1,2,3,4,5]. As a core and indispensable passive component in RF front-end architectures, tunable attenuators fulfill critical functions including signal power leveling, receiver dynamic range optimization, and impedance mismatch compensation. These functions directly govern the signal integrity and overall energy efficiency of the entire system [6,7,8].

**Figure 1:**
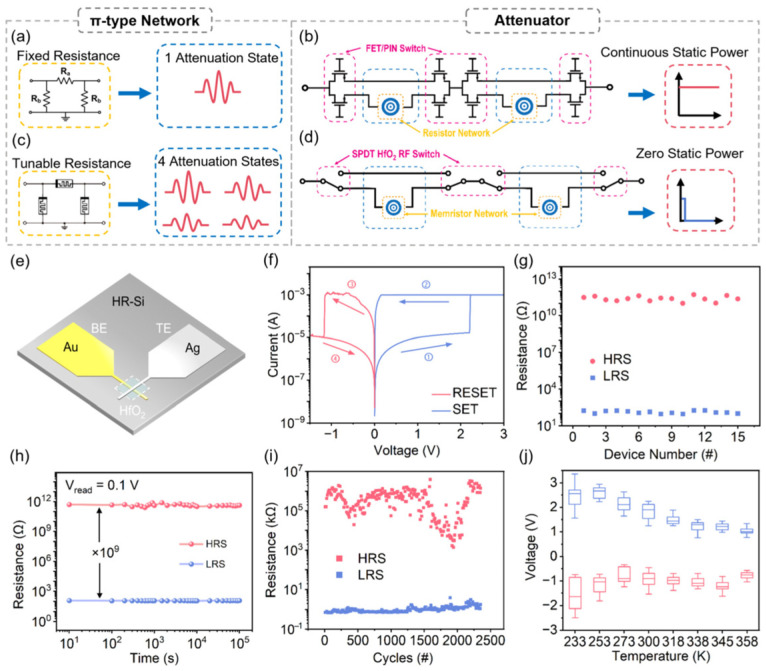
DC switching characteristics of HfO_2_-based memristor. (**a**) Conventional π-type attenuation network based on fixed resistors. (**b**) Architecture of the conventional tunable attenuator based on FET/PIN solid-state switches and fixed resistor networks. (**c**) The proposed tunable π-type attenuation network constructed by HfO_2_-based memristors with tunable resistance, enabling 4 discrete attenuation states via resistance programming. (**d**) Architecture of the proposed zero-static-power reconfigurable attenuator based on HfO_2_ memristor single-pole double-throw (SPDT) RF switches and programmable memristor network. (**e**) Schematic illustration of an HfO_2_-based crossbar memristor with a gold bottom electrode (BE) and a silver top electrode (TE). (**f**) Representative *I*-*V* curve of bipolar non-volatile resistive switching effect in Au/HfO_2_/Ag device with 4 × 4 μm^2^ overlap area and SET *I*_CC_ of 1 mA. (**g**) Device-to-device uniformity of resistive switching states for 15 identical Au/HfO_2_/Ag devices under bilayer photoresist process and sputtered HfO_2_. (**h**) The retention performance of HRS and LRS was measured at room temperature with a small bias of +0.1 V. A retention time of 10^5^ s was achieved, verifying the reliable and stable non-volatile behavior of the device. (**i**) Endurance characteristics of the device with 2300 continuous switching cycles (# means cycles number) at a SET voltage of 4 V and a RESET voltage of −3.5 V at a readout voltage of 0.1 V. (**j**) Temperature dependence of the programming voltages. The SET voltage (red) decreases with increasing temperature, whereas the RESET voltage (blue) exhibits no obvious temperature dependence.

A π-type attenuator is a type of passive attenuator that uses a resistive network circuit (Figure 1a). Due to the fixed resistance, the π-type attenuator only outputs 1 attenuation state. To achieve tunable attenuators, the conventional methods adopt the cascading of solid-state switches (PIN diodes or GaAs field-effect transistors, FETs) with fixed resistor networks (Figure 1b), yet this architecture suffers from two inherent bottlenecks [9,10]. On one hand, to break through the limitation of fixed resistor networks, the program of solid-state switches requires continuous direct current (DC) bias to sustain their operating states. This leads to significant static power consumption, as well as severe insertion loss and linearity degradation at high frequencies [11]. On the other hand, the hybrid integration of discrete switches and resistive networks introduces pronounced parasitic effects, which not only hinder further device miniaturization but also incur additional signal loss in the gigahertz range [12]. These drawbacks render conventional solutions unable to satisfy the stringent requirements of next-generation RF systems for compact footprint, ultra-low power consumption, and wideband operation [13].

Conductive bridging random access memory, which achieves non-volatile resistive switching through the reversible formation and rupture of nanoscale conductive filaments (CF) in a simple metal-insulator-metal (MIM) structure, Refs. [14,15] has emerged as a promising alternative for next-generation RF devices due to the zero static power consumption [16,17,18,19]. Among various functional materials, HfO_2_ stands out with its high tolerance to oxygen vacancies and excellent pathways for metal ion migration, which is a promising candidate to fabricate stable and repeatable non-volatile memristor with ultra-low multistate ON-state resistance (*R*_ON_). The replacement of both fixed resistors and RF switches with HfO_2_-based memristor in a π-type tunable attenuator shows the potential to overcome the inherent bottlenecks (Figure 1c,d). However, the utilization of reconfigurable *R*_ON_ ability in memristor for realizing switching-attenuation integrated multistate attenuators is still lacking.

In this article, we demonstrate a non-volatile HfO_2_-based memristor as an RF switch with tunable *R*_ON_, enabling an all-memristor attenuator with reconfigurable multistate attenuation. We first elucidate the electrochemical mechanism underlying CF formation, and establish the quantitative mapping between *I*_CC_, *R*_ON_, and RF insertion loss, as well as between device dimensions, OFF-state capacitance (*C*_OFF_), and isolation. The fabricated memristor exhibits stable non-volatile bipolar resistive switching, with *R*_ON_ continuously tunable from 5.8 Ω to 197.5 Ω in Au/HfO_2_/Ag and from 4.1 Ω to 22 Ω in Ag/HfO_2_/Ag via *I*_CC_ modulation. We demonstrate the wideband RF performance of the device from 10 MHz to 43.5 GHz through on-wafer S-parameter measurements. We reveal the dependence of insertion loss (from −8.24 dB to −0.53 dB) on ON-state resistance (from 197.5 Ω to 5.8 Ω) and the OFF-state isolation (from −26.8 dB to −23.8 dB) on device geometry (4 μm^2^ to 25 μm^2^). Finally, we demonstrate a reconfigurable attenuator based on our multi-state memristor with tunable low-resistance levels in a cascaded 2-unit configuration. The attenuator achieves 12 discrete attenuation levels from 2 dB to 24 dB and a return loss better than 10 dB across the full band. This work provides a systematic device physics framework for HfO_2_-based RF switches, together with a facile, monolithically integrable solution enabling programmable multistate attenuators.

## 2. Materials and Methods

### 2.1. Device Fabrication and Characterization

Initially, the HfO_2_ memristor and RF switches were fabricated on a high-resistance silicon (HR-Si) substrate via bilayer photolithography (AZ5214 and LOR10A photoresist). Memristors were patterned into crossbar array structures, while RF switches were configured in a ground-signal-ground (GSG) coplanar waveguide (CPW) layout. The bottom electrodes of Au (35 nm)/Cr (5 nm) and Ag (150 nm)/Cr (5 nm) were deposited by thermal evaporation. After bottom electrode deposition, the photoresist was removed in N-Methylpyrrolidone (NMP), followed by acetone and isopropanol rinses, and finally dried with nitrogen (N_2_). Next, the HfO_2_ switching layer was patterned by photolithography, followed by sputter deposition of a 30 nm HfO_2_ film and lift-off in acetone. The deposition was performed using a HfO_2_ target (99.99% purity) in a pure Ar atmosphere at a flow rate of 37 sccm. The RF power was set to 100 W, yielding a deposition rate of 0.067 nm/s over a duration of 450 s. Finally, the top electrode of Ag (150 nm) was deposited by thermal evaporation after which any residual photoresist was completely removed by sequential cleaning in NMP (30 min), acetone (10 min) and isopropanol (10 min), followed by N_2_ drying. The thickness of the HfO_2_ switching layer was determined by atomic force microscopy (AFM) (Bruker Dimension ICON, Bruker, Billerica, MA, USA). The structure of the RF switches was examined by scanning electron microscopy (SEM) (Zeiss Sigma 300, Zeiss, Jena, TH, Germany).

### 2.2. DC Measurement

The electrical characteristics of the devices were measured at room temperature using a Keithley 2600 semiconductor parameter analyzer (Keithley, Solon, OH, USA), including resistive switching, endurance, and retention characteristics. The temperature-dependent measurements were performed under vacuum conditions over a temperature range of 233 K to 358 K, with the chamber pressure maintained at 10^−6^ Torr. During the measurements, the voltage was applied to the top electrode, while the bottom electrode was grounded.

### 2.3. RF Measurement

The 100 μm-pitch GSG probes (DSK-F40-GSG-100, DesignCon Technology Co., Ltd., Shenzhen, China) and the Keysight N5234B PNA-L vector network analyzer (VNA) (Keithley, Solon, OH, USA) were used for on-wafer measurements from 10 MHz to 43.5 GHz. Before measurement, a two-port short-open-load-through (SOLT) calibration was performed using a commercial substrate to mitigate parasitic effects from the cables, probes, and VNA. Following calibration, the GSG probes were contacted to the pads of the device under test with a probe station, enabling direct characterization by the VNA.

## 3. Results and Discussion

### 3.1. DC Switching Characteristics of Au/HfO_2_/Ag Memristors

Au/HfO_2_/Ag crossbar memristors (Figure 1e) were implemented to obtain the ultra-low and multistate R_ON_. The low resistance in conductive-bridge memristor makes it own the advantages for RF switch compared with oxygen-vacancy, and proton-based memristor [20]. HfO_2_ was fabricated by magnetron sputtering to introduce sufficient defects, which are utilized as the active layer in memristors and RF switching devices. Due to the high tolerance for oxygen vacancies in the sputtered HfO_2_ layer, excellent pathways were obtained for Ag ion migration. In addition, the contact quality at the HfO_2_/Ag interface was also optimized by the bilayer-photoresist photolithography (BPP) process. This BPP process creates an atomically smooth, residue-free interface with uniform electric field distribution, enabling synchronous growth of multiple parallel and uniform Ag CFs. This controlled CF dynamics eliminates switching randomness and ensures consistent low-resistance state (LRS) values under identical programming conditions. Consequently, the device provides the controllability needed for accurate multi-state programming in RF attenuators [21].

Based on the above-mentioned fabrication process, systematic DC electrical characterizations were performed, revealing their intrinsic bipolar and non-volatile resistive switching characteristics. The devices can be SET from the high-resistance state (HRS) to the LRS via a positive voltage sweep, and RESET from the LRS back to the HRS via a negative voltage sweep. The inherent non-volatility of this resistive switching enables the devices to sustain their programmed *R*_ON_ persistently without continuous electrical biasing [22]. Figure 1f illustrates the current-voltage (*I*-*V*) characteristics of the 4 × 4 μm^2^ Au/HfO_2_/Ag device fabricated through the BPP process. We acquired this data via a sequential voltage sweep on the Ag top electrode over the range of 0 V to +3.0 V, back to 0 V, then to −1.5 V, and finally back to 0 V, while maintaining the Au bottom electrode at ground potential throughout the test. A compliance current (*I*_CC_) of 1 mA was set for the SET process to avert permanent breakdown of the device. At a sweep voltage of ~2.2 V, the current increases sharply to the compliance value, indicating the SET transition from HRS to LRS via the formation of Ag CFs across the HfO_2_ layer. When the voltage sweeps back from +3.0 V to 0 V, the device maintains the LRS, demonstrating its non-volatile characteristic. The RESET transition occurs at −1.1 V, where the current drops sharply as the CFs rupture via Joule heating, returning the device to the HRS. As shown in Figure 1g, the Au/HfO_2_/Ag device achieves tightly concentrated *R*_ON_ (100–180 Ω) with small device-to-device variation. As a control experiment, we fabricated an Au/HfO_2_/Ag memristor by the monolayer-photoresist photolithography (MPP) process. Compared with memristors via the BPP process, devices via the MPP process show extremely dispersed LRS resistance spanning ~80 Ω to 850 Ω (shown in Figure S1). Another control experiment adopts the atomic layer deposition (ALD) to fabricate dense HfO_2_ in the Au/HfO_2_/Ag memristors. The high *R*_ON_ of ~1200 Ω was obtained due to the difficulty of Ag ion migration in this dense HfO_2_ film (Figure S2). These results demonstrate the successful optimization of our Au/HfO_2_/Ag memristor.

As verified by the retention tests in Figure 1h, the device achieves a stable ON/OFF ratio of ~10^9^. Under a read voltage of +0.1 V at room temperature, both the LRS and HRS remained stable for 10^5^ s, which attests to the device’s excellent stability. Moreover, the endurance of the threshold-switching behavior has been proved by applying pulsed voltage stresses, showing no degradation of the response resistance after more than 1500 cycles, as shown in Figure 1i. The low, robust and nonvolatile ON state (LRS) resistance was obtained, which can be used for improving RF insertion loss. Furthermore, an additional endurance test for 10^5^ cycles under low switch ratio conditions (Figure S3) of the Au/HfO_2_/Ag device indicated that it is suited for programmable attenuator applications requiring stable tuning. Figure 1j illustrates the dependence of the programming voltages on ambient temperature. The SET voltage shows a clear decreasing trend as the temperature increases from 233 K to 345 K, indicating that filament formation becomes easier at elevated temperatures due to thermally activated Ag ion migration. Ag ion diffusion is thermally dominated, which is driven by the concentration gradient (Figure S4). The dominant Ag ion drift is ensured after the competition between drift and diffusion, achieving non-volatile memory states due to the optimized defect density in sputtered HfO_2_. In contrast, the RESET voltage remains relatively stable across the same temperature range, suggesting that the rupture of the CF is primarily governed by Joule heating and is insensitive to ambient temperature variations [23]. These observations align with the electrochemical mechanism of filament formation and thermal-assisted rupture in memristor devices.

In addition, given that Ag electrodes may promote more efficient CF formation and potentially yield lower on-state resistance, we also examined Ag/HfO_2_/Ag devices for comparative evaluation. We characterized the DC switching performance of Ag/HfO_2_/Ag devices with identical geometries and test conditions (Figure S5). The Ag/HfO_2_/Ag device exhibited similar bipolar non-volatile resistive switching behavior and extremely low SET and RESET voltages. However, its retention and endurance characteristics were inferior to those of the Au/HfO_2_/Ag device.

### 3.2. RF Characterization of HfO_2_ Switches

**Figure 2:**
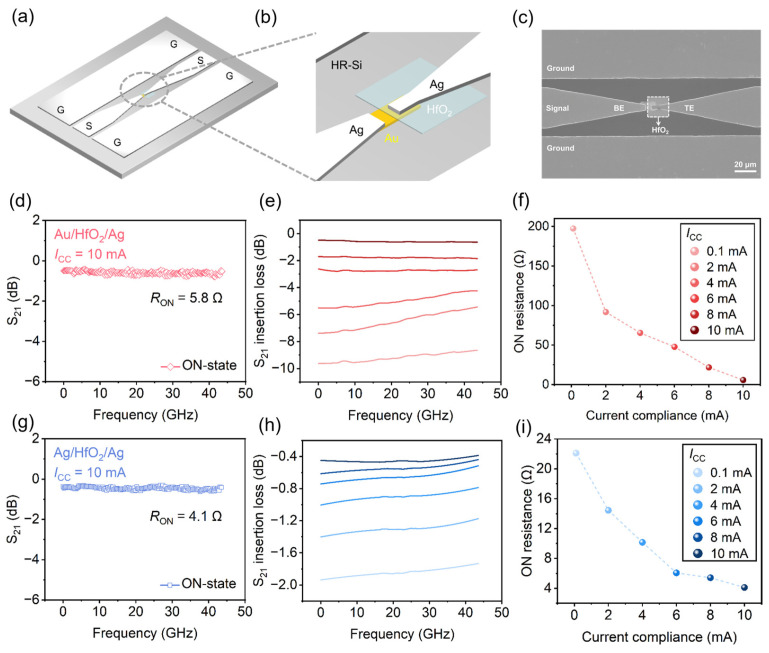
Structural characterization of the fabricated HfO_2_-based RF switch and ON-state characteristics of Au/HfO_2_/Ag and Ag/HfO_2_/Ag switches. (**a**) 3D schematic illustration of the fabricated Au/HfO_2_/Ag RF switch with a ground-signal-ground (GSG) coplanar waveguide configuration (CPW) on a high-resistivity silicon (HR-Si) substrate. (**b**) Enlarged schematic view of the vertical Au/HfO_2_/Ag metal-insulator-metal (MIM) stack structure of the memristor switch. (**c**) Top-view scanning electron microscopy (SEM) image of the fabricated RF switch, with the HfO_2_ active region marked and a scale bar of 20 μm. (**d**,**g**) Frequency-dependent ON-state S_21_ of devices under 10 mA *I*_CC_ from 10 MHz to 43.5 GHz, with extracted ON-state resistance. (**e**,**h**) S_21_ insertion loss across 10 MHz~43.5 GHz under varying *I*_CC_ levels. (**f**,**i**) Extracted ON-state resistance scaling with different *I*_CC_ for the two device configurations.

Based on the ultra-low *R*_ON_ in Au/HfO_2_/Ag, we further characterized the RF switching performance and its reconfigurability. Figure 2a,b represents 3D schematic illustrations of the fabricated Au/HfO_2_/Ag vertical RF switch, which adopts a GSG CPW configuration for RF signal transmission and a vertical MIM stack for resistive switching. Figure 2c presents an SEM image of the fabricated RF switch, integrated with a GSG CPW for RF signal transmission. Figure S6 shows an AFM image and the corresponding thickness profile of a 4 × 4 μm^2^ vertical Au/HfO_2_/Ag sandwich structure fabricated on an HR-Si substrate, with the HfO_2_ layer having a thickness of approximately 30 nm. The fabrication process flow of the HfO_2_ RF switch is detailed in Figure S7.

The RF performance of the fabricated HfO_2_-based resistive switches was characterized via on-wafer S-parameter measurements for both ON and OFF states using a VNA. A full on-wafer SOLT calibration was performed prior to testing to de-embed parasitic contributions from the test cables, RF probes, and probe station, enabling accurate extraction of the intrinsic RF characteristics of the devices. An equivalent lumped element circuit (Figure S8) was employed to extract the key performance parameters, namely *R*_ON_ and *C*_OFF_.

For the Au/HfO_2_/Ag switches, the ON-state RF performance and *I*_CC_ -dependent tunability of the HfO_2_-based RF switches were evaluated. For the Au/HfO_2_/Ag switches, the measured ON-state de-embedded S_21_ under *I*_CC_ of 10 mA is shown in Figure 2d. The S_21_ maintains a near-constant value of −0.53 dB across the frequency range of 10 MHz–43.5 GHz, exhibiting a low insertion loss characteristic for RF signal transmission. An *R*_ON_ of 5.8 Ω was extracted for the device from the measured S-parameters (see Note S1 for detailed calculations). The dependence of ON-state insertion loss on *I*_CC_ is presented in Figure 2e, where the S_21_ insertion loss decreases from −8.24 dB to −0.53 dB as the *I*_CC_ increases from 0.1 mA to 10 mA. This trend is directly correlated to the linear scaling of *R*_ON_ with *I*_CC_ (Figure 2f), where *R*_ON_ decreases continuously from 197.5 Ω at 0.1 mA to 5.8 Ω at 10 mA, demonstrating defined tunability of the device’s conductive state via *I*_CC_ modulation, which is the core enabling technology for the monolithic integrated attenuator design in this work.

This *R_ON_*–*I*_CC_ dependence can be understood from the conductive filament growth dynamics. During the SET process, the *I*_CC_ limits the total cross-sectional area or the number of parallel Ag filaments formed across the HfO_2_ layer. A higher *I*_CC_ allows a thicker or more numerous filament bundle to stabilize before the current-limiting circuit engages, thereby reducing the overall *R_ON_*. The quasi-linear scaling observed in Figure 2f is consistent with this picture, as the filament conductance scales approximately with its cross-sectional area. Consequently, the ON-state insertion loss, which is dominated by the ohmic loss of the series switch, can be quantitatively linked to *R*_ON_ through the relation IL (dB) ≈ −20 *lg* [1 + *R*_ON_/(2*Z*_0_)] for a well-matched 50 Ω system. This expression captures the measured trend from −8.24 dB at *R*_ON_ = 197.5 Ω to −0.53 dB at *R*_ON_ = 5.8 Ω, bridging the DC programming condition and the resulting RF transmission performance in a straightforward manner.

For comparison, the ON-state RF performance of Ag/HfO_2_/Ag switches with identical test conditions is shown in Figure 2g–i. For the Ag/HfO_2_/Ag device under 10 mA *I*_CC_ (Figure 2g), the ON-state de-embedded S_21_ remains stable at −0.36 dB across 10MHz-43.5 GHz, with an extracted *R*_ON_ of 4.1 Ω, which is lower than that of the Au/HfO_2_/Ag counterpart under the same operating conditions. The ON-state insertion loss of the Ag/HfO_2_/Ag switches decreases with increasing *I*_CC_, with the S_21_ insertion loss reducing from −1.9 dB to −0.36 dB as the *I*_CC_ increases from 0.1 mA to 10 mA (Figure 2h). The *R*_ON_ of the Ag/HfO_2_/Ag switches also scales linearly with *I*_CC_, ranging from 22 Ω at 0.1 mA to 4.1 Ω at 10 mA (Figure 2i). Moreover, the different *I*_CC_ sweep curves for the two devices are shown in Figure S9.

Collectively, these results confirm that for a given *I*_CC_, the Ag/HfO_2_/Ag stack exhibits a lower *R*_ON_ than the Au/HfO_2_/Ag stack. With two active Ag electrodes, Ag can be supplied from either side, depending on the bias polarity [24]. In this case, the low LRS resistance was obtained. This lower *R*_ON_ directly accounts for the reduced on-state insertion loss of the Ag/HfO_2_/Ag device at the same operating frequency, which can be well explained by the series resistance loss model for RF transmission lines: a lower *R*_ON_ reduces the ohmic loss of the transmitted RF signal, thus improving the insertion loss performance [25,26]. This finding is also consistent with the widely accepted consensus in the field that the on-state insertion loss of RF switches is dominantly determined by their *R*_ON_ [18]. In addition, the Au/HfO_2_/Ag stack structure offers a significantly wider *R*_ON_ tuning range and better long-term switching stability with the aid of the established Schottky barriers at the Au/HfO_2_ interfaces. For the stability of intermediate resistance states, a higher *I*_CC_ produces thicker Ag filaments with reduced interfacial energy, making them thermodynamically more resistant to spontaneous rupture. As demonstrated by the retention measurements (Figure S10), all six intermediate resistance states used in our attenuator design (programmed at *I*_CC_ = 0.1 to 10 mA) exhibited negligible drift over 10^5^ s at room temperature, confirming their suitability for reliable reconfigurable attenuator operation. For reconfigurable attenuator applications, the wide-range, high-precision *R*_ON_ tuning capability of Au/HfO_2_/Ag devices is a primary consideration, as it enables a wider attenuation tuning range and more discrete attenuation levels [8].

**Figure 3:**
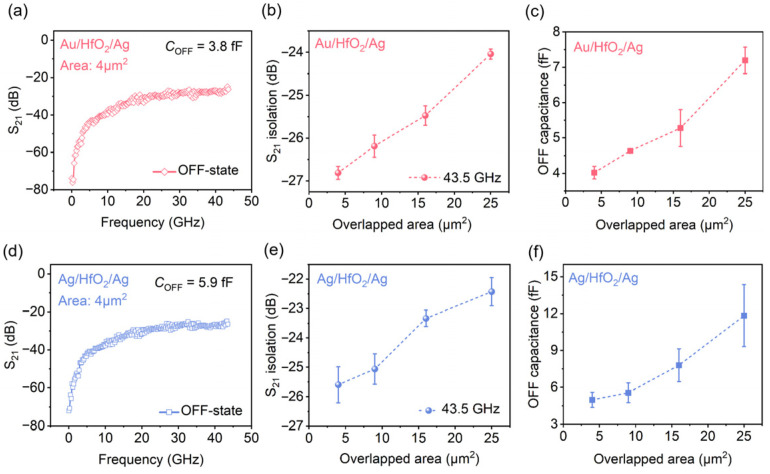
OFF-state characteristics of Au/HfO_2_/Ag and Ag/HfO_2_/Ag switches. (**a**,**d**) Frequency-dependent OFF-state S_21_ of 4 μm^2^ devices from 10 MHz to 43.5 GHz, with extracted OFF-state capacitance. (**b**,**e**) S_21_ isolation at 43.5 GHz versus device overlapped area. (**c**,**f**) OFF-state capacitance scaling with overlapped area for the two device configurations.

In addition, the measured OFF-state de-embedded forward transmission coefficient (S_21_) of the 4 μm^2^ device is shown in Figure 3a under the same test configuration as the ON-state characterization. From 10 MHz to 43.5 GHz, the S_21_ increases from −75 dB to −26.8 dB. This reveals a frequency-dependent roll-off in isolation. This effect stems from intrinsic capacitive coupling in the MIM stack during the high-resistance OFF state. A *C*_OFF_ of 3.8 fF was extracted for this 4 μm^2^ device from the measured S-parameters. The dependence of OFF-state isolation on device geometry is presented in Figure 3b, where the isolation at 43.5 GHz decreases monotonically from −26.8 dB to −23.8 dB as the device overlapped area increases from 4 μm^2^ to 25 μm^2^, demonstrating that larger device dimensions degrade OFF-state isolation. This trend is directly correlated to the linear scaling of *C*_OFF_ with the overlapped area (Figure 3c), where *C*_OFF_ increases from ~4 fF to ~7.2 fF across the same area range, consistent with the fundamental scaling behavior of parallel-plate capacitors.

For direct comparison, the OFF-state RF performance of Ag/HfO_2_/Ag switches with identical device geometries is shown in Figure 3d–f. For the 4 μm^2^ Ag/HfO_2_/Ag device (Figure 3d), the OFF-state de-embedded S_21_ increases from −75 dB at 10 MHz to −25.8 dB at 43.5 GHz, with an extracted *C*_OFF_ of 5.9 fF, which is higher than that of the Au/HfO_2_/Ag counterpart with the same area. As with the Au/HfO_2_/Ag devices, the OFF-state isolation of the Ag/HfO_2_/Ag switches at 43.5 GHz degrades with increasing overlapped area, decreasing from −25.8 dB to −22.2 dB as the area increases from 4 μm^2^ to 25 μm^2^ (Figure 3e). The *C*_OFF_ of the Ag/HfO_2_/Ag switches also scales linearly with the overlapped area, ranging from ~5 fF at 4 μm^2^ to ~11.5 fF at 25 μm^2^ (Figure 3f). Collectively, the isolation performance of RF switches in the OFF-state is primarily dictated by the *C*_OFF_ [27]. According to the parallel-plate capacitor theory, a larger electrode overlap area leads to a higher capacitance, thereby degrading the isolation of HfO_2_-based RF switches [17,28,29]. These results confirm that for a given device area, the Ag/HfO_2_/Ag stack exhibits a larger *C*_OFF_ than the Au/HfO_2_/Ag stack, which directly explains the reduced OFF-state isolation of the Ag/HfO_2_/Ag switches at the same operating frequency.

Based on the extracted *R*_ON_ and *C*_OFF_ values, we further calculated the cutoff frequency (*f*_c_), a critical figure of merit for RF switches defined as *f*_c_ = 1/2π*R*_ON_*C*_OFF_, which represents the maximum operating frequency where the switch maintains acceptable ON/OFF performance. For the Au/HfO_2_/Ag switch with *R*_ON_ = 5.8 Ω and *C*_OFF_ = 3.8 fF, the extrapolated *f*_c_ reaches ~7.2 THz. For the Ag/HfO_2_/Ag counterpart with *R*_ON_ = 4.1 Ω and *C*_OFF_ = 5.9 fF, the *f*_c_ is ~6.6 THz. Both values are orders of magnitude higher than the measured upper frequency limit of 43.5 GHz in this work, demonstrating the exceptionally high-frequency potential of HfO_2_-based memristor switches for millimeter-wave and even terahertz applications.

### 3.3. Tunable Attenuators for Signal Processing

**Figure 4:**
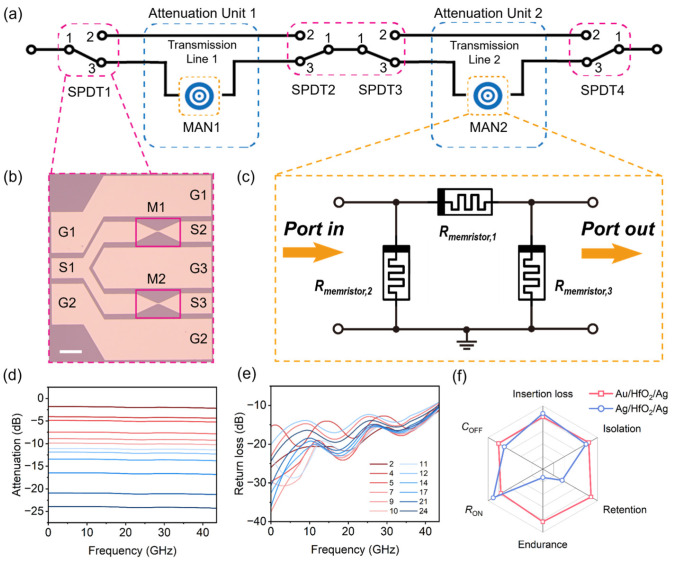
Schematic diagram and performance of the attenuator based on the HfO_2_ memristor network and RF switch. (**a**) Schematic of the 2-unit cascaded reconfigurable attenuator architecture integrated with single-pole double-throw (SPDT) memristor switches and memristor attenuation networks (MANs). (**b**) Optical microscope image of the fabricated SPDT RF switch based on HfO_2_ memristors with a ground-signal-ground (GSG) coplanar waveguide (CPW) configuration. The scale bar is 50 μm. (**c**) Circuit topology of the symmetric π-type MAN, constructed by three identical HfO_2_-based memristors. (**d**,**e**) Simulated performance of the attenuator: (**d**) attenuation and (**e**) return loss over the 10 MHz-43.5 GHz range. (**f**) Radar chart of Au/HfO_2_/Ag and Ag/HfO_2_/Ag device, the position closer to the outer edge of the radar plot represents the better performance in the corresponding dimension.

Leveraging the non-volatile, wide-range *R*_ON_ tunability of the fabricated Au/HfO_2_/Ag RF switches, we propose an all-memristor reconfigurable attenuator based on symmetric π-type topology, realizing monolithic integration of switching and attenuation functions without any additional passive components or bias networks. Figure 4a shows the architecture of the 2-unit cascaded attenuator. The attenuator adopts a path-switchable cascaded topology, consisting of 4 single-pole double-throw (SPDT) memristor switches and 2 identical memristor attenuation networks (MANs, Attenuation Units 1 and 2). The SPDT switches (optical image in Figure 4b) control the RF signal path. When an SPDT selects the through path (port 1–2), the RF signal bypasses the corresponding MAN with near-zero insertion loss. When it selects the attenuation path (port 1–3), the signal passes through the MAN to achieve the preset attenuation. The MAN unit is the symmetric π-type all-memristor topology shown in Figure 4c. Different from conventional π-type attenuators relying on fixed resistors, this topology is constructed entirely by three identical Au/HfO_2_/Ag memristors: one memristor in the series arm (R_memristor,1_) and two identical memristors in the shunt arms (R_memristor,2_ and R_memristor,3_). By precisely tuning the *I*_CC_ during the SET process, the *R*_ON_ of each memristor can be programmed to the target value, thus realizing accurate setting of the attenuation amount of the π-type network.

Based on the measured RF characteristics of the fabricated devices, we systematically quantified the attenuation performance of the proposed π-type architecture. According to microwave circuit theory, a quantitative relationship between the attenuation value and the resistance in the MAN is established (see Note S2 for details). Based on this, we can determine the four attainable attenuation levels from the memristor resistance values set via the *I*_CC_. By setting the series memristor (R_memoristor,1_) to the minimum programmable *R*_ON_ of 5.8 Ω and configuring the two parallel memristors (R_memoristor,2_ and R_memoristor,3_) to HRS, the desired 2 dB attenuation can be achieved. Detailed memristor configurations for another 3 attenuation levels are shown in Table S1. Thus, a single π-type MAN unit can achieve four discrete attenuation levels of 2 dB, 5 dB, 9 dB and 12 dB. Then, we further adopt a 2-unit cascaded architecture based on the proposed π-type unit. Benefiting from the consistent input/output impedance of each π-type unit (50 Ω), the total attenuation of the cascaded system realizes linear superposition of the attenuation of each individual unit. This is achieved without accumulating impedance mismatches or degrading high-frequency performance. The simulated RF responses are illustrated in Figure 4d,e, showing 12 different attenuation states with an attenuation tuning range of 2 dB to 24 dB across the full 10 MHz to 43.5 GHz band. The return loss is better than 10 dB for all attenuation states across the entire operating band, demonstrating excellent impedance matching. This path-switchable design enables the attenuator to achieve 12 discrete attenuation levels from 2 dB to 24 dB, only by tuning the resistance state of the memristors and the switching state of the SPDT switches, without hardware modification. It offers the advantages of a compact structure, zero static power consumption, broadband stability, and high reconfigurability.

Meanwhile, to minimize parasitic effects in practical circuits, several strategies were adopted. At the device level, the vertical MIM structure reduces parasitic series inductance compared to lateral designs, the optimized 4 μm^2^ device area achieves a low OFF-state capacitance of 3.8 fF, and the high-resistivity silicon substrate (ρ > 10 kΩ·cm) suppresses substrate coupling losses. At the circuit level, the symmetric π-type topology inherently provides 50 Ω matching, while the GSG CPW layout with optimized dimensions ensures controlled impedance interconnections. All RF measurements were preceded by full on-wafer SOLT calibration to de-embed probe pad and cable parasitics.

Compared with conventional attenuator architectures, the proposed all-memristor π-type topology exhibits three distinct advantages for next-generation communication applications. First, the attenuator uses only identical two-terminal memristor switches. This eliminates the need for the separate switch matrices and resistor networks found in traditional designs. As a result, the device footprint is reduced, enabling ultra-high monolithic integration. Second, the zero static power consumption characteristic, derived from the non-volatile nature of memristor switches, is a critical improvement over conventional solid-state switch-based attenuators that require continuous DC bias. Third, the proposed architecture supports discrete attenuation tuning via *I*_CC_ regulation without hardware modification, exhibiting excellent reconfigurability.

For all-memristor reconfigurable attenuators, Au/HfO_2_/Ag and Ag/HfO_2_/Ag memristors with unique performance can enrich the design flexibility. We selected six core performance dimensions of RF switches, including insertion loss, isolation, retention, endurance, on-state resistance, and off-state capacitance, and performed a normalized comparison of the performance of Au/HfO_2_/Ag and Ag/HfO_2_/Ag devices through the radar plot (Figure 4f). The Au/HfO_2_/Ag device demonstrates superior performance in terms of stability and durability, offering a stable and controllable programmable resistance state. The Ag/HfO_2_/Ag device exhibits superior RF performance with ultra-low *R*_on_ and low insertion loss, desirable for RF transmission lines. Our work provides a reliable device foundation for the subsequent design and implementation of all-memristor reconfigurable attenuators. As summarized in Table S2, our Au/HfO_2_/Ag and Ag/HfO_2_/Ag memristor RF switches present clear advantages over both oxide-based and 2D material-based RF switches operating at frequencies below 120 GHz [8,17,19,27,30,31,32,33,34,35,36]. They achieve drastically higher switching ratios, better isolation, lower insertion loss and higher cutoff frequency than other oxide devices, while outperforming 2D devices in isolation and endurance with equally low insertion loss. Furthermore, Table S3 compares the performance of the all-memristor tunable attenuator with other reported tunable attenuators, Refs. [7,8,13,37,38,39] highlighting its significant advantages of zero static power consumption due to the non-volatile nature of HfO_2_ switches, as well as higher integration density achieved with only 2 cascaded units.

## 4. Conclusions

In summary, we have systematically investigated HfO_2_-based two-port RF switches designed for integrated attenuator applications. The Au/HfO_2_/Ag devices demonstrate stable non-volatile bipolar resistive switching with a high ON/OFF ratio of ~10^9^ and reliable retention of 10^5^ s. A key finding is the quantitative relationship between *I*_CC_, ON-state resistance, and RF insertion loss. The *R*_ON_ can be continuously tuned from 5.8 Ω to 197.5 Ω, providing the analog programmability essential for reconfigurable attenuation. On-wafer S-parameter measurements from 10 MHz to 43.5 GHz further reveal the scaling laws linking device geometry to OFF-state isolation and *I*_CC_ to ON-state insertion loss. We proposed a π-type cascaded attenuator architecture that achieves 12 discrete attenuation levels from 2 dB to 24 dB, enabled solely by *I*_CC_ tuning without additional passive components or static power consumption. These results not only provide a comprehensive understanding of the material-device-RF performance correlation in HfO_2_-based memristor switches, but also present a simple, monolithically integrable pathway toward low-power, reconfigurable RF front-ends for next-generation communication systems.

Beyond insertion loss and isolation, practical RF front-end applications also require consideration of switching speed, linearity, and power handling. The intrinsic switching in HfO_2_-based conductive bridge memristors is governed by nanosecond-scale Ag ion migration, suggesting inherently fast reconfiguration capability. Regarding linearity, the ON-state metallic filament behaves as a passive linear resistor under small-signal excitation, unlike semiconductor switches that introduce nonlinear capacitances. Future studies will systematically characterize these parameters under high-speed pulsed and large-signal conditions, and explore device engineering strategies, such as adjusting the HfO_2_ thickness, to extend power handling toward transmitter-level requirements.

## Data Availability

The original contributions presented in this study are included in the article/Supplementary Materials. Further inquiries can be directed to the corresponding author.

## Supplementary Materials

The following supporting information can be downloaded at: https://www.mdpi.com/article/10.3390/nano16100605/s1. Note S1: Calculation of resistance (*R*) and capacitance (*C*) from S-parameters; Note S2: Calculation of resistance values in π-type network RF attenuators; Figure S1: (a) Typical bipolar resistive switching *I-V* curve of the Au/HfO_2_/Ag device fabricated by single-layer photoresist process. (b) LRS resistance distribution of 15 identical devices fabricated by single-layer and bilayer photoresist processes, with *I*_CC_ of 1 mA; Figure S2: *R*_ON_ of Au/HfO_2_/Ag memristors with ALD grown film; Figure S3: Endurance of Au/HfO_2_/Ag resistive switching devices under low switching ratio conditions; Figure S4: The schematic diagram of Ag ion drift and diffusion; Figure S5: Device profile and electrical characteristic of Ag/HfO_2_/Ag RF switch; Figure S6: AFM analysis of Au/HfO_2_/Ag structure; Figure S7: Schematic illustration of the fabrication process for the Au/HfO_2_/Ag RF switch; Figure S8: Lumped element equivalent circuit of HfO_2_ RF switch; Figure S9: *I-V* characteristics of HfO_2_ switches under different *I*_CC_; Figure S10: Retention characteristics of intermediate resistance states in Au/HfO_2_/Ag memristors programmed at different *I*; Table S1: Parameters of π-type network topologies corresponding to different attenuation levels; Table S2: Performance comparison of memristor-based RF switches operating below 120 GHz; Table S3: Performance comparison of tunable RF attenuators. References [40,41,42] are cited in the Supplementary Materials.
