# Supplementary material for: HfO2-Based Reconfigurable Radio Frequency Switches for All-Memristor Multistate Attenuator
Source: Nanomaterials (Basel). 2026 May 15;16(10):605. doi: 10.3390/nano16100605 (PMC13209425; doi:10.3390/nano16100605)
Supplement: Supplementary file 1 [file nanomaterials-16-00605-s001.zip › nanomaterials-4309081-supplementary.pdf]

HfO_2 Based Reconfigurable Radio Frequency Switches for All-Memristor Multistate Attenuator

Yuanyuan Zhou[1,4†], Yan Wu[1†], Quan Yang[1,*], Weiran Cai[1], Xiaowei Zhang[4], Xiaolong Cai[2, 3,*], Chenglin Du[2, 3], Yuda Zhao[1,*]

[1] College of Integrated Circuits, ZJU-Hangzhou Global Scientific and Technological Innovation Center, Zhejiang University, 38 Zheda Road, Hangzhou 310027, People's Republic of China

[2] State Key Laboratory of Mobile Network and Mobile Multimedia Technology, Shenzhen 518055, People's Republic of China

[3] ZTE Corporation, Shenzhen 518057, People's Republic of China

[4] Faculty of Electrical Engineering and Computer Science, Ningbo University, Ningbo 315211, People's Republic of China

*Correspondence: yangquan@zju.edu.cn; cai.xiaolong@zte.com.cn; yudazhao@zju.edu.cn

[†] These authors contribute equally

Note S1: Calculation of resistance (R) and capacitance (C) from S-parameters

In microwave engineering, scattering parameters (S-parameters) measured by a Vector Network Analyzer (VNA) are typically output as magnitude and phase values. To analyze the intrinsic electrical properties of a two-port network (such as an RF switch), these S-parameters must be converted to complex rectangular form, then translated to impedance (Z) or directly to admittance (Y) parameters. Below is the standardized workflow, detailed calculation formulas for this conversion, and the method to extract the on-state resistance (R) and off-state capacitance (C) of RF switches.

1. Preprocessing of measured S-parameters to complex rectangular form

A single S-parameter can be expressed in polar form as:

$$S = M \cdot [cos(\theta) + j \cdot sin(\theta)](1)$$

where M is the linear magnitude of the S-parameter, θ is the phase angle in radians, and j is the imaginary unit ($j^2 = -1$). To obtain the complex rectangular form required for subsequent network analysis from raw VNA measurements, a set of standard conversions are implemented in sequence: the linear magnitude M is converted from the dB-form measurement result through the relation $M = 10^{\frac{dB\ value}{20}}$, the phase angle θ is converted from degrees to radians via the formula $\theta\ (\text{rad}) = \theta\ (\text{deg}) \times \frac{\pi}{180}$, and the complex S-parameter is finally decomposed into rectangular form, with its real part calculated as $\text{Re(S)} = M \cdot \cos(\theta)$ and its imaginary part calculated as $\text{Im(S)} = \text{M} \cdot \sin(\theta)$.[1]

2. Standard conversion from S-parameters to admittance (Y) parameters

For a two-port network, the S-parameter matrix is defined as:

$$S = \begin{bmatrix} S_{11} & S_{12} \\ S_{21} & S_{22} \end{bmatrix} (2)$$

where S_{11} = input reflection coefficient, S_{22} = output reflection coefficient, S_{21} = forward transmission coefficient, and S_{12} = reverse transmission coefficient.

The admittance matrix Y, which describes the frequency-domain current-voltage relationship of the two-port network, can be directly calculated from the S-parameter matrix (without intermediate Z-parameter conversion) using the standard formula validated in mainstream microwave engineering references.[2] The full matrix conversion is:

$$Y = \frac{Y_0}{\Delta_S} \begin{bmatrix} (1 - S_{11})(1 + S_{22}) + S_{12}S_{21} & -2S_{12} \\ -2S_{21} & (1 + S_{11})(1 - S_{22}) + S_{12}S_{21} \end{bmatrix} (3)$$

where Z_0 is the characteristic impedance of the RF measurement system (standard value of 50 Ω for most microwave setups), $Y_0 = 1/Z_0$ is the characteristic admittance of the system, Δ_S is the core determinant term for S-to-Y conversion, defined as:

$$\Delta_S = (1 + S_{11})(1 + S_{22}) - S_{12}S_{21} \quad (4)$$

Expanding Equation (3), the individual elements of the Y-parameter matrix are explicitly given by:

$$Y_{11} = Y_0 \cdot \frac{(1 - S_{11})(1 + S_{22}) + S_{12}S_{21}}{\Delta_S} \quad (5)$$

$$Y_{12} = Y_0 \cdot \frac{-2S_{12}}{\Delta_S} \quad (6)$$

$$Y_{21} = Y_0 \cdot \frac{-2S_{21}}{\Delta_S} \quad (7)$$

$$Y_{22} = Y_0 \cdot \frac{(1 + S_{11})(1 - S_{22}) + S_{12}S_{21}}{\Delta_S} \quad (8)$$

These formulas fully account for both direct and cross-port coupling in the two-port network. For reciprocal passive networks (e.g., RF switches), the reciprocity theorem holds $S_{12} = S_{21}$, which simplifies calculations as $Y_{12} = Y_{21}$.

3. RF switch ON/OFF state analysis: extraction of R and C

The key performance metrics of a series-type RF switch are its on-state series resistance R (which dominates insertion loss in the ON state) and off-state parasitic capacitance C (which dominates isolation in the OFF state). Both metrics can be directly extracted from the complex transfer admittance Y_{12}.

In the OFF state, the RF switch behaves as a small parasitic capacitor, with its admittance dominated by the reactive (imaginary) component. The off-state capacitance is calculated as:

$$C = \frac{Im(Y_{12})}{2\pi f} \quad (9)$$

where f is the operating frequency in hertz (Hz).

In the ON state, the RF switch behaves as a low-value series resistor, with its admittance dominated by the conductive (real) component. The on-state resistance is calculated as:

$$R = \frac{1}{Re(Y_{12})} \quad (10)$$

Note S2: Calculation of resistance values in π-type network RF attenuators

A π-type attenuation network can be modeled as a two-port network. In the low-frequency range, the relationships between the input and output voltages and currents at the ports are determined using the ABCD linear network parameters. In the high-frequency range, the input-output voltage relationship of the attenuation network is determined using the S-parameters of the linear network.[3] At high frequencies, the input and output impedances of the attenuation network circuit are equal to the characteristic impedance (Z_0 = 50 Ω). From the perspective of a symmetrical structure, there is $S_{21} = S_{12}$ and $S_{11} = S_{22}$. When the attenuation network circuit is impedance-matched with the system, there is $S_{11} = 0$.[4] Therefore, the S-parameters of the π-type attenuation network is:

$$\begin{bmatrix} S_{11} & S_{12} \\ S_{21} & S_{22} \end{bmatrix} = \begin{bmatrix} 0 & S_{12} \\ S_{21} & 0 \end{bmatrix} \quad (1)$$

The ABCD metric of the π-type attenuation network is:

$$\begin{bmatrix} A & B \\ C & D \end{bmatrix} = \begin{bmatrix} 1 + \frac{R_a}{R_b} & R_a \\ \frac{2R_b + R_a}{R_b^2} & -1 - \frac{R_a}{R_b} \end{bmatrix} \quad (2)$$

According to the conversion relationship between the S-parameters and the ABCD metric, the following can be obtained:

$$A = 1 + \frac{R_a}{R_b} = \frac{1 + S_{21}^2}{2S_{21}} \quad (3)$$

$$B = R_a = Z_0 \cdot \frac{1 - S_{21}^2}{2S_{21}} \quad (4)$$

$$C = \frac{2R_b + R_a}{R_b^2} = \frac{1}{Z_0} \cdot \frac{1 - S_{21}^2}{2S_{21}} \quad (5)$$

$$D = -1 - \frac{R_a}{R_b} = \frac{1 + S_{21}^2}{2S_{21}} \quad (6)$$

The values of resistors R_a and R_b in a π-type attenuator network can be derived from equations (3) and (4):

$$R_a = Z_0 \cdot \frac{1 - 10^{-\frac{A_{dB}}{10}}}{2 \times 10^{-\frac{A_{dB}}{20}}} \quad (7)$$

$$R_b = Z_0 \cdot \frac{1 + 10^{-\frac{A_{dB}}{20}}}{1 - 10^{-\frac{A_{dB}}{20}}} \quad (8)$$

where A_{dB} represents the attenuation (in dB), Z_0 represents the characteristic impedance (50 Ω), then the magnitude of attenuation is $S_{21} = 10^{-\frac{dB}{20}}$.

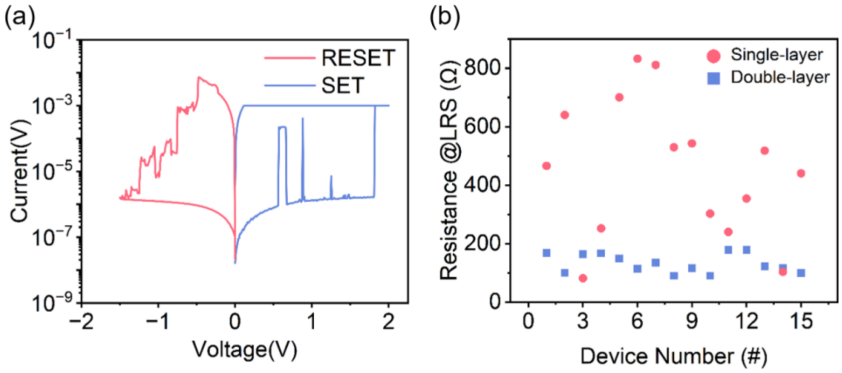

Figure S1: (**a**) Typical bipolar resistive switching *I-V* curve of the Au/HfO_2/Ag device fabricated by single-layer photoresist process. (**b**) LRS resistance distribution of 15 identical devices fabricated by single-layer and bilayer photoresist processes, with I_{CC} of 1 mA.

Figure S2 compares the resistive switching characteristics and device uniformity of Au/HfO_2/Ag devices fabricated by single-layer and bilayer photoresist processes, with the performance difference governed by interface quality modulation of CF dynamics. As shown in **Figure S3**(a), the single-layer photoresist device exhibits severe stochastic bipolar switching: the SET process (blue curve) shows multiple irregular current spikes without a clear threshold voltage, while the RESET process (red curve) displays dramatic current fluctuations. This uncontrolled behavior originates from the poor metal-HfO_2 interface of the single-layer process, where photoresist residues, edge burrs, and high roughness induce localized electric field concentration. This restricts Ag^+ nucleation to random defect sites, forming inhomogeneous, dendritic CFs that grow and rupture asynchronously, leading to erratic *I-V* behavior.

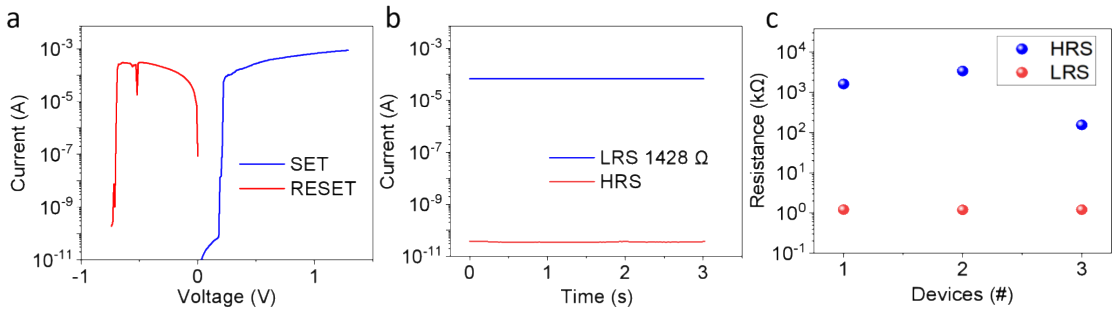

Figure S2: R_{ON} of Au/HfO_2/Ag memristors with ALD grown film. (**a**) DC curve *I-V* characteristics, (**b**) *I-t* characteristics under read voltage 0.1 V, showing the R_{ON} of 1428 Ω, (c) Other three memristors with the R_{ON} of 1220 Ω, 1200 Ω and 1215 Ω.

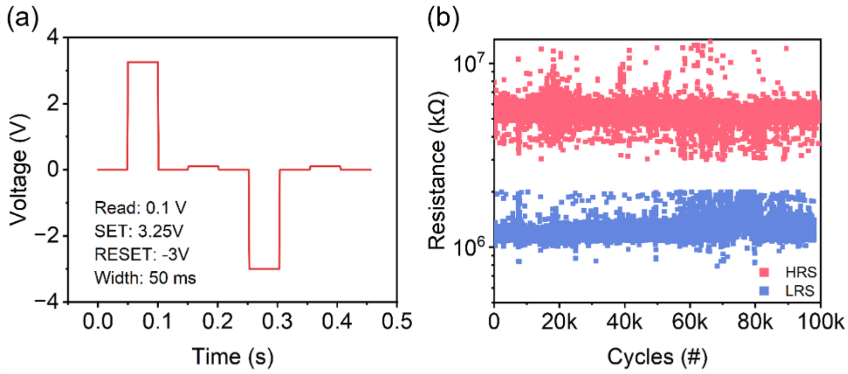

Figure S3: Endurance of Au/HfO_2/Ag resistive switching devices under low switching ratio conditions. (a) Voltage pulse sequence used for resistive switching operations, including a 3.25 V SET pulse, a −3 V RESET pulse (both with a pulse width of 50 ms), and a 0.1 V read pulse for non-destructive resistance state readout. (b) Resistance evolution curves for the high-resistance state (HRS, red) and low-resistance state (LRS, blue) during 100,000 consecutive SET/RESET switching cycles, with all resistance values measured at a 0.1 V readout voltage.

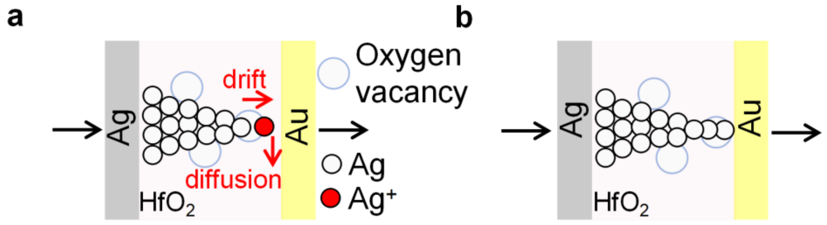

Figure S4: a The schematic diagram of Ag ion drift and diffusion. b The schematic diagram of forming Ag filament.

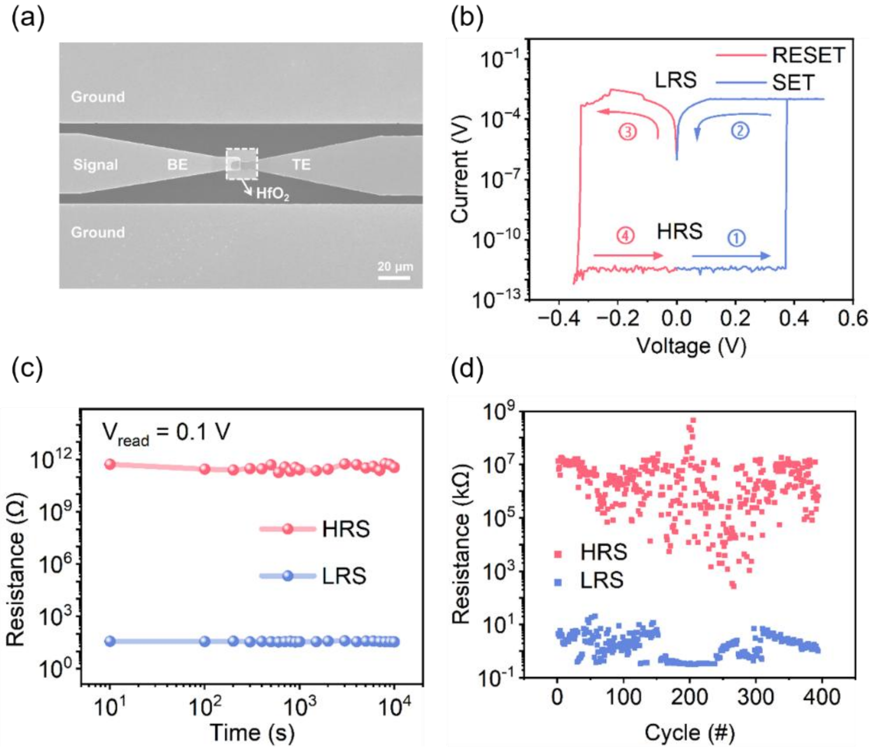

Figure S5: Device profile and electrical characteristic of Ag/HfO_2/Ag RF switch. (a) Scanning electron microscopy (SEM) image of the 4 × 4 μm^2 Ag/HfO_2/Ag switch and the dashed square marks the area where the HfO_2 was deposited. (b) Representative I–V curve of bipolar non-volatile resistive switching effect in Ag/HfO_2/Ag device with 4 × 4 μm^2 overlap area and SET compliance current of 1 mA. Step 1: The voltage is swept from 0 to +0.5 V. At ~0.38 V, a sharp current increase to the compliance value, indicating the SET transition from HRS to LRS. Step 2: Decreasing the voltage from +0.5 to 0 V maintains the LRS, demonstrating non-volatile characteristics. Step 3: The voltage decreases from 0 to -0.4 V. The sharp drop in current approximately -0.34 V shows the RESET process from LRS to HRS. Step 4: The voltage returns back to 0 V. (c) The retention performance of HRS and LRS states measured at room temperature with a small bias of +0.1 V. (d) Switching characteristics of the memristive RF switch under DC pulse driving.

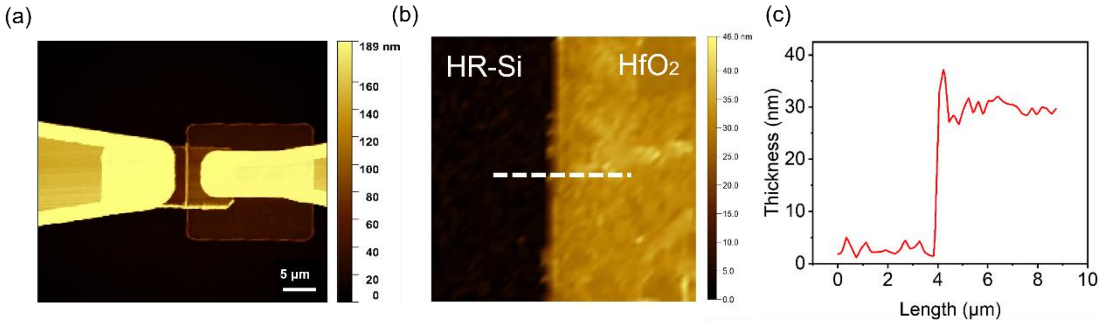

Figure S6: Atomic force microscopy (AFM) analysis of Au/HfO_2/Ag structure. (a) AFM image of the Au/HfO_2/Ag structure with the overlapped area of 4×4 μm^2 (b) 2D AFM images and thickness profiles of a sputtered HfO_2 layer on a high-resistance silicon (HR-Si) substrate. (c) The thickness profile of the HfO_2 switching layer shows a thickness of approximately 30 nm.

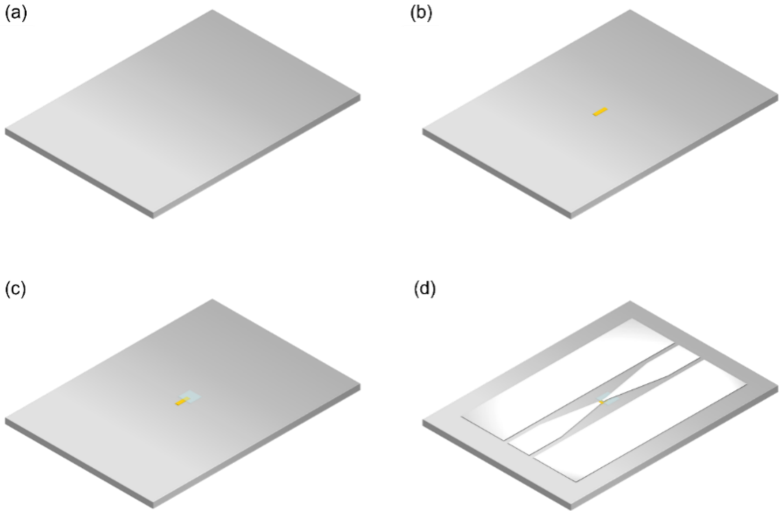

Figure S7: Schematic illustration of the fabrication process for the Au/HfO_2/Ag radio frequency (RF) switch. (a) The high-resistance silicon (HR-Si) substrate was rinsed with acetone and dried with the nitrogen spray gun. (b) Results of photolithography and thermal evaporation processes for the bottom electrode (Au). (c) Results following photolithography and magnetron sputtering processes on the switching layer (HfO_2). (d) Results of the photolithography and thermal evaporation processes for the bottom electrode (Ag).

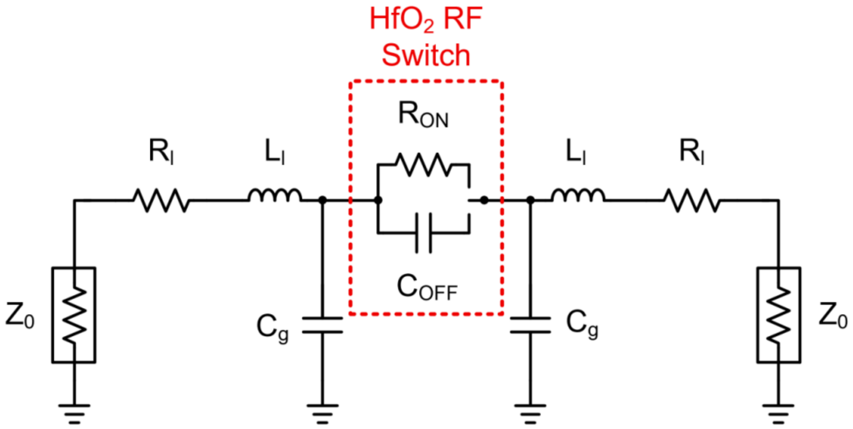

Figure S8: Lumped element equivalent circuit of HfO_2 RF switch. The schematic illustrates line resistance (R_l), line inductance (L_l), characteristic impedance (Z_0), coupling capacitance (C_g) in parallel with ground, and either the switch's OFF-state capacitance (C_{OFF}) or ON-state resistance (R_{ON}).[5] The components in the circuit represent interconnection parasitic parameters, which can be eliminated through a two-step de-embedding method using shorted and opened switch configurations.

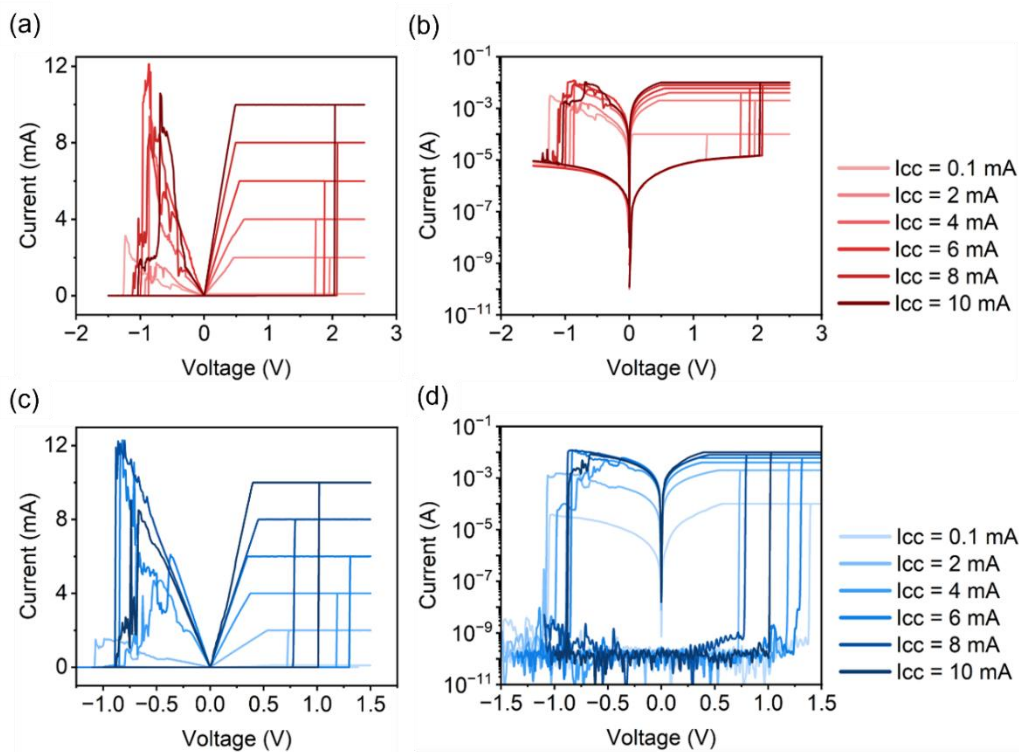

Figure S9: *I-V* characteristics of HfO_2 switches under different I_{CC}. (a, b) *I-V* characteristics of bipolar resistive switching behavior in Au/HfO_2/Ag devices, shown on a linear scale and a logarithmic scale, respectively. DC cycling measurements were performed at bias currents of 0.1, 2, 4, 6, 8, and 10 mA. (c, d) *I-V* characteristics of bipolar resistive switching behavior in Ag/HfO_2/Ag devices, shown on a linear scale and a logarithmic scale, respectively.

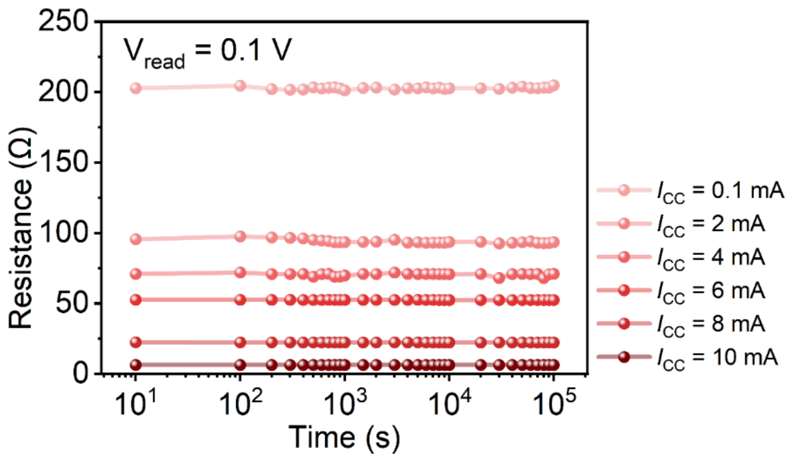

Figure S10: Retention characteristics of intermediate resistance states in Au/HfO2/Ag memristors programmed at different compliance currents (ICC = 0.1 mA to 10 mA) under a read voltage of 0.1 V at room temperature. All six intermediate resistance states exhibit excellent non-volatile stability over the entire 105 s test period, which satisfies the precision requirements for programmable RF attenuator applications.

Table S1: Parameters of π-type network topologies corresponding to different attenuation levels.

Memristor	2dB	5dB	9dB	12dB
$R_{memristor,1}$	5.8 Ω	21.75 Ω	47.8 Ω	91.84 Ω
$R_{memristor,2}$ $R_{memristor,3}$	HRS	197.46 Ω	91.84 Ω	65.45 Ω
Z_{in}	55.8 Ω	46.98 Ω	42.81 Ω	42.37 Ω

Table S2: Performance comparison of memristor-based RF switches operating below 120 GHz.

Device type	Structure	Control voltage (V)	Switching ratio	Endurance	Retention at room temperature (s)	Insertion loss (dB)	Isolation (dB)	Bandwidth (GHz)	Cutoff frequency (THz)	Ref.
Oxide	Ni/HfO_2/Ni	−1 to 0.5	10^4	10^6	10^4	2	13	110	0.84	[1]
	Au/VO_x/Ag	−1 to 2.5	10^7	10^5	2×10^5	0.45	20	67	4.5	[5]
	Al/Ti/HfO_2/W	−1 to 0.5	10	130	N/A	2.1	10	50	0.0081	[6]
	**Au/HfO_2/Ag**	**−1.1 to 2.2**	**10^9**	**2300 (on/off ratio 10^5)<br>10^5 (on/off ratio 10)**	**10^5**	**0.53**	**26.8**	**43.5**	**7.2**	**This work**
	**Ag/HfO_2/Ag**	**−0.34 to 0.38**	**10^{10}**	**400**	**10^4**	**0.36**	**25.8**	**43.5**	**6.6**	
Oxide-2D heterostructure	Au/WO_{3-x}/WSe_2/Ag	−1.5 to 2.5	10^3	32	10^5	0.7	30	67	25.2	[7]
	Ag/HfO_2/MoS_2/Pt	−0.6 to 0.7	1.5×10^3	50	N/A	1.3	9.2	43.5	N/A	[8]
	Ag/ZnO/MoS_2/Pt	−1 to 2.5	10^3	N/A	N/A	2	10.4	30	N/A	[9]
Polymer	Au/pV3D3/Au	−0.64 to 3.37	10^5	1600	4.1×10^5	0.28	19	20	5.38	[10]
	Cu/Nafion/Au	−2.5 to 10	10^3	175	N/A	0.68	11.55	43.5	N/A	[11]
2D	Au/MoS_2/Au	−0.7 to 1.4	10^3	20	10^4	0.3	20	50	5.8	[12]
	Ag/MoS_2/Ag	−1.1 to 1.75	10^6	300	10^7	0.7	10	12	0.56	[13]
	Au/h-BN/Au	−0.5 to 3	10^5	2000	10^5	0.9	10	120	N/A	[14]

Table S3: Performance comparison of tunable RF attenuators

Tuning Component	Bandwidth (GHz)	Attenuation range(dB)	Number of tuning states	Number of cascaded units	Static power consumption	Ref.
Varactor Diode	5	2.1-32.7	Analog	9	Required	[15]
PCM GeTe	26-34	0-24.1	9	4	Required	[16]
Graphene	2	1.7-8.4	Analog	2	Required	[17]
GaN HEMT	26.5-40	0-7	8	3	Required	[18]
Liquid Crystal	32.5-35	0-13	8	8	Near 0	[19]
2D WO_{3-x}/WSe_2 heterostructure	34	3-33	11	4	Near 0	[7]
**HfO_2 switch**	**43.5**	**2-24**	**12**	**2**	**Near 0**	**This work**

References

1. Chen, S.-C.; Yang, Y.-T.; Tseng, Y.-C.; Chiou, K.-D.; Huang, P.-W.; Chih, J.-H.; Liu, H.-Y.; Chou, T.-T.; Jhang, Y.-Y.; Chen, C.-W.; et al. HfO_2 Memristor-Based Flexible Radio Frequency Switches. *ACS Nano* **2025**, *19*, 704–711, doi:10.1021/acsnano.4c11846.
2. Pozar, D. M. Microwave Engineering: Theory and Techniques. John Wiley & Sons. 2021.
3. Otto, S.; Bettray, A.; Solbach, K. A Distributed Attenuator for K-Band Using Standard SMD Thin-Film Chip Resistors. In Proceedings of the 2009 Asia Pacific Microwave Conference; IEEE: Singapore, Singapore, December 2009; pp. 2148–2151.
4. Yiqin Sun; Lei Li; Han Lin; Zhiyuan Yu; Mian Huang; Lixi Wan Attenuators Using Thin Film Resistors for RF Application. In Proceedings of the 2008 International Conference on Electronic Packaging Technology & High Density Packaging; IEEE: Shanghai, July 2008; pp. 1–3.
5. Seo, D.; Kim, D.; Ryu, J.; Pyo, C.; Lee, S.; Yoon, T.; Kim, M. VOx-based Non-volatile Radio-frequency Switches for Reconfigurable Filter. *Adv. Sci.* **2025**, e01989, doi:10.1002/advs.202501989.
6. Amarilla, T.; González, M.B.; Campabadal, F.; Nafria, M.; Crespo-Yepes, A.; Paco, P. de; Verdú, J. Fabrication and Characterization of Al/Ti/HfO2/W Memristor-Based RF Switches. *IEEE Microw. Wirel. Technol. Lett.* **2026**, 1–4, doi:10.1109/LMWT.2026.3678995.
7. Cao, A.; Li, S.; Xu, Z.; Zhang, Z.; Xu, X.; Cui, A.; Jiang, K.; Shang, L.; Li, Y.; Zhu, L.; et al. Miniaturized Reconfigurable WO3–x/WSe2-Based 2D Radio-Frequency Switches with Integrated Attenuator and Phase Shifter for next-Gen Communication. *Adv Funct Mater* **2025**, e10452, doi:https://doi.org/10.1002/adfm.202510452.
8. Zhang, T.; Liu, H.; Sheng, L.; Shen, Y.; Tang, X.; Li, X.; Shen, G.; Qin, F.; Yao, J.; Wang, Z.; et al. A Non-Volatile Radio Frequency Switch Based on an Ag/HfO2/MoS2/Pt Structure Memristor. *Chip* **2026**, 100199, doi:10.1016/j.chip.2026.100199.
9. Song, Y.; Zhu, G.-Y. Low Voltage and High Speed Operation Non-Volatile RF Switch Based on Metal Oxide Memristor. In Proceedings of the 2025 IEEE MTT-S International Wireless Symposium (IWS); IEEE: Xi'an, China, May 19 2025; pp. 1–3.
10. Park, S.; Pyo, C.; Yu, J.H.; Lee, S.; Kim, M.J.; Kim, M. Thermally Robust Polymer-Based Analogue Switch for Flexible mmWave Application. *Adv. Funct. Mater.* **2026**, *36*, e27948, doi:10.1002/adfm.202527948.
11. Shen, Y.; Sheng, L.; Song, Y.; Zhang, T.; Cui, B.; Wang, Z.; Cai, Z.; Tang, X.; Xu, R.; Shen, G.; et al. A High Speed Response Nafion-Based Non-Volatile RF Switch. *Appl. Phys. Lett.* **2025**, *126*, 242103, doi:10.1063/5.0270011.
12. Kim, M.; Ge, R.; Wu, X.; Lan, X.; Tice, J.; Lee, J.C.; Akinwande, D. Zero-Static Power Radio-Frequency Switches Based on MoS2 Atomristors. *Nat. Commun.* **2018**, *9*, 2524, doi:10.1038/s41467-018-04934-x.
13. Xiao, X.; Peng, Z.; Zhang, Z.; Zhou, X.; Liu, X.; Liu, Y.; Wang, J.; Li, H.; Novoselov, K.S.; Casiraghi, C.; et al. Fully Printed Zero-Static Power MoS2 Switch Coded Reconfigurable Graphene Metasurface for RF/Microwave Electromagnetic Wave Manipulation and Control. *Nat. Commun.* **2024**, *15*, 10591, doi:10.1038/s41467-024-54900-z.

14. Pazos, S.; Shen, Y.; Zhang, H.; Verdú, J.; Fontana, A.; Zheng, W.; Yuan, Y.; Alharbi, O.; Ping, Y.; Guerrero, E.; et al. Memristive Circuits Based on Multilayer Hexagonal Boron Nitride for Millimetre-Wave Radiofrequency Applications. *Nat. Electron.* **2024**, *7*, 557–566, doi:10.1038/s41928-024-01192-2.
15. Yuan, Y.; Chen, S.J.; Fumeaux, C. Transmission-Type Varactor-Based Tunable Attenuator. *IEEE Trans. Microw. Theory Tech.* **2024**, *72*, 5082–5094, doi:10.1109/TMTT.2024.3370305.
16. Singh, T.; Mansour, R.R. Scalable mmWave Non-Volatile Phase Change GeTe-Based Compact Monolithically Integrated Wideband Digital Switched Attenuator. *IEEE Trans. Electron Devices* **2021**, *68*, 2306–2312, doi:10.1109/TED.2021.3069729.
17. Wu, B.; Fan, C.; Feng, X.; Zhao, Y.-T.; Ning, J.; Wang, D.; Su, T. Dynamically Tunable Filtering Attenuator Based on Graphene Integrated Microstrip Resonators. *IEEE Trans. Microw. Theory Tech.* **2020**, *68*, 5271–5278, doi:10.1109/TMTT.2020.3017197.
18. Jang, S.; Yang, J.; Lee, J.; Park, C. A Ka-Band 3-Bit GaN Digital Step Attenuator Using Phase Compensation Method. *IEEE Access* **2023**, *11*, 125835–125843, doi:10.1109/ACCESS.2023.3326818.
19. Tu, L.; Meng, F.-Y.; Ding, C.; Han, J.-Q.; Liu, Y.-H.; Liu, C.; Wei, W. A Liquid Crystal-Based Tunable Millimeter-Wave Attenuator Design. *IEEE Trans. Microw. Theory Tech.* **2025**, *73*, 4523–4533, doi:10.1109/TMTT.2025.3548471.
